# Metabolic Profiling of Plasma in Different Calving Body Condition Score Cows Using an Untargeted Liquid Chromatography-Mass Spectrometry Metabolomics Approach

**DOI:** 10.3390/ani10091709

**Published:** 2020-09-21

**Authors:** Jian Wang, Chuang Zhang, Qingyao Zhao, Congcong Li, Shuang Jin, Xianhong Gu

**Affiliations:** State Key Laboratory of Animal Nutrition, Institute of Animal Sciences, Chinese Academy of Agricultural Sciences, Beijing 100193, China; wangjian_1884@163.com (J.W.); 18241653656@163.com (C.Z.); zhaoqingyao63@163.com (Q.Z.); congcongli1988@sina.com (C.L.); shuangjinjs@163.com (S.J.)

**Keywords:** calving body condition score, metabolomics, lysophosphatidylcholines, phosphatidylethanolamine, cow

## Abstract

**Simple Summary:**

Over-conditioning around calving predisposes cows to a higher risk of postpartum metabolic disorders and diseases. Using untargeted liquid chromatography-mass spectrometry, seven metabolites were found that were significantly correlated to high body condition, as well as to classic metabolic parameters indicating changes in lipid metabolism and inflammation. These seven metabolites include six distinct lysophosphatidylcholines and a phosphatidylethanolamine. By investigating the depth of metabolic changes in the plasma of over-conditioned fresh cows, this study may provide a metabolic basis for the diseases associated with over-conditioning.

**Abstract:**

This study was undertaken to identify metabolite differences in plasma of dairy cows with a normal or high calving body condition score (CBCS), using untargeted liquid chromatography-mass spectrometry (LC-MS) metabolomics. Sixteen multiparous dairy cows were assigned to one of two groups based on CBCS (0 to 5 scale): Normal group (NBCS, 3.25 ≤ BCS ≤ 3.5, *n* = 8), and high BCS group (HBCS, BCS ≥ 4, *n* = 8). Plasma samples were collected for metabolomics analysis and evaluation of biomarkers of lipid metabolism (nonesterified fatty acid (NEFA) and β-hydroxybutyrate (BHB)), and cytokines (leptin, adiponectin, tumor necrosis factor–α (TNF-α) and interleukin 6 (IL-6)). A total of 23 differential metabolites were identified, and functional analyses were performed using the Kyoto Encyclopedia of Genes and Genomes (KEGG) pathways. Among these metabolites, the concentrations of six lysophosphatidylcholines and one phosphatidylethanolamine, were lower in the HBCS group than in the NBCS group (*p* < 0.01). Furthermore, these metabolites were involved in these four pathways, among others: glycerophospholipid metabolism, retrograde endocannabinoid signaling, autophagy, and glycosylphosphatidylinositol (GPI)-anchor biosynthesis (*p* < 0.05). In addition, plasma concentrations of leptin (*p* = 0.06) and TNF-α (*p* = 0.08) tended to be greater while adiponectin (*p* = 0.09) lower in HBCS cows than in NBCS cows. The concentrations of NEFA, BHB, or IL-6 did not differ between NBCS and HBCS groups. More importantly, based on the results of the Spearman’s correlation analysis, the seven important metabolites were negatively correlated with indices of lipid metabolisms, proinflammatory cytokines, and leptin, but positively correlated with adiponectin. These results demonstrate that CBCS has a measurable impact on the plasma metabolic profile, even when NEFA and BHB are not different. In addition, the identified differential metabolites were significantly correlated to lipid metabolism and inflammation in the over-conditioned fresh cows, which are expected to render a metabolic basis for the diseases associated with over-conditioned dry cows.

## 1. Introduction

Body condition score (BCS) is a subjective measurement system widely used in dairy farms for evaluating body fat reserve [1,2], and it is well known that BCS can be a clue to the nutrition status in dairy herd [3]. In dairy cows, overfeeding leads to over-condition around calving. Previous studies have shown that over-conditioning precalving decreases dry matter intake pre- and post-calving, and after calving leads to greater body condition loss and greater negative energy balance [4,5], as well as predispose cows to greater incidence of postpartum metabolic disorders and different diseases [6], such as ketosis, fatty liver, clinical mastitis and milk fever [3]. Therefore, the fat cows are more likely to fail to adapt their metabolism to the onset of lactation, partially contributed to decreased milk yield and increased treatments, thus causing great economic losses to the dairy farm.

More recently, metabolomics are being used to study obesity and metabolic disorders in humans [7,8], dogs [9,10], and mice [11,12]. Metabolomic analysis has revealed biomarkers for the detection of shifts in obesity, as well as the identification of animals for treatment of obesity-related disease [13]. The CBCS is of great significance to the health and welfare of dairy cows, but its effect on plasma metabolomic profiling has been ignored. It is known that untargeted metabolomics aims to examine all detectable compounds in samples [14]. Hence, the measurement of a large number of small molecular metabolites by untargeted metabolomics using LC-MS may help to illuminate the metabolic alternations for the increased health disorders associated with over-conditioned at calving in dairy cows.

In the present study, as the most commonly used metabolic variables in the metabolic monitoring of dairy cows [15], the concentrations of NEFA and BHB were measured, which are significantly correlated with BCS of cows [16,17]. In addition, we also focus on four different cytokines, including leptin and adiponectin, which serve to control energy homeostasis, as well as TNF-α and IL-6, the important proinflammatory cytokines [15]. It has been documented that the concentrations of these two metabolic variables and four cytokines are linked with obesity and obesity-related disorders [15,17,18]. Therefore, the objective of this study was to investigate (1) the metabolites that might be early indicators of altered plasma metabolism in fresh cows with a normal or high CBCS and (2) the relationships between the newly identified metabolites and classic metabolic parameters.

## 2. Material and Methods

All the experimental protocols and animal care were performed in accordance with the standards established by the Institute of Animal Science, Chinese Academy of Agricultural Sciences (Approval number: IAS2018-7).

This study was conducted at a large-scale farm, Hebei Shounong Modern Agricultural Technology Co. Ltd. (Hebei, China). During the study, the cows were certified clinically healthy without metabolic disease history and were housed in a free stall barn with free access to freshwater. A total mixed ration (TMR) was provided three times a day, at 07:00, 13:30, and 20:00 h, allowing a 5% orts. Cows were fed the same diet during the close-up dry period (three weeks before the expected parturition date) that could meet the nutritional needs of dairy cows recommended by NRC (2001). The TMR, containing 1.7% ground corn, 4.5% wheat bran, 4.5% soybean meal, 1.4% rapeseed meal, 4.5% distillers dried grains with solubles, 3.6% apple pomace, 2.0% whole cottonseed, 13.6% oat hay, 59.1% corn silage, 4.5% sodium cyclamate, and 0.6% premix, was formulated to provide 3.9% ether extract, 14.7% CP, 34.87% NDF, 22.25% ADF and 1.55 mcal/kg of energy (NE_L_).

### 2.1. Experimental Design and Samples Collection

The BCS was evaluated by two trained professional persons who had four years of BCS-evaluating experience. The BCS evaluation system was based on the method described by Edmonson et al. [2] on a scale of 1 (emaciated) to 5 (fat) with increments of 0.25. Sixteen multiparous Chinese Holstein cows that had similar age (month: 61.2 ± 4.9 (SD)), gestation length (day: 275.7 ± 7.4 (SD)), previous 305-d mature equivalent milk yield (11175 ± 665 Kg) and parity (2.81 ± 0.4 (SD)), but with two levels of CBCS: A normal group (NBCS, 3.25 ≤ BCS ≤ 3.5, *n* = 8), and a high BCS group (HBCS, BCS ≥ 4, *n* = 8). The average CBCS for NBCS and HBCS groups were 3.34 ± 0.13 (SD) and 4.06 ± 0.12 (SD), respectively.

Blood samples were taken immediately after calving, but before colostrum milking, from the coccygeal blood vessels into vacutainer tubes (BD Biosciences, San Jose, CA) containing heparin. Blood samples were placed on ice for transport to the laboratory where the plasma was separated by centrifugation at 3000× *g* for 15 min at 4 °C. Plasma was stored at −80 °C until further laboratory procedures.

### 2.2. LC-MS Metabolomics Analysis

Sixteen samples were thawed at room temperature, and 100 μL of each sample was transferred precisely into 1.5 mL centrifuge tubes. Then a 400 μL extraction solution (methanol/acetonitrile, 1:1, *v*/*v*) was added to samples. After vortexing, three ultrasonic extractions of 10 min on the ice were performed. The samples were allowed to stand at −20 °C for 30 min. The supernatant was separated by centrifugation at 13,000× *g* for 15 min using a refrigerated centrifuge at 4 °C. Then, the supernatant was lyophilized using an LNG-T88 rapid centrifugal concentration dryer (Taicang Huamei Biochemical Instrument factory, Taicang, China). After lyophilization, 100 μL of reconstitution solution (acetonitrile/water, 1:1, *v*/*v*) was added before being transferred to a vial for LC-MS analyses.

All samples were analyzed using an ultra-high pressure liquid chromatography (UPLC) coupled with a Triple quadrupole time-of-flight (TOF) system (ABSCIEX-Triple TOF 5600; AB SCIEX, Framingham, MA, USA). The chromatographic separation was performed at 40 °C with a column of BEH C18 (100 mm × 2.1 mm, 1.7 µm, Waters, Milford, MA, USA). The injection volume of the pretreated plasma samples was 20 µL. The two mobile phase consisted of 0.1% formic acid aqueous solution (solvent A) and acetonitrile/isopropanol (*v*/*v*, 1:1) with 0.1% formic acid (solvent B). The flow rate was 0.40 mL/min and mobile phase (A:B) elution gradient was as follows: 95%:5% for 0 min, 80%:20% for 0 to 3.0 min, 5%:95% for 3.0 to 9.0 min, 5%:95% for 9.0 to 13.0 min,95%:5% for 13.0 to 13.1 min, and 95%:5% for 13.1 to 16.0 min. The mass spectral analysis (MS) and tandem mass spectral analysis (MS/MS) were performed on a Triple TOF mass spectrometer with an electrospray ionization source operated both in positive and negative-ion mode. The capillary voltage, injection voltage, and collision energy were 1.0 kV, 40 V, and 6 eV, respectively. The ion source temperature and desolvation temperatures were 120 °C and 500 °C, respectively. The nitrogen flow was 900 L/h, mass data were collected between mass to charge ratio (*m/z*) 50–1000, and the instrument resolution was 30,000. In order to evaluate the stability of the analytical system, quality control (QC) samples prepared by mixing aliquots of all plasma samples were injected at regular intervals throughout the analytical run.

### 2.3. Measurement of Classical Metabolic Parameters

Plasma concentrations of NEFA (Cat No. JYM0103Bo, Wuhan jiyinmei Biotechnology Co., Ltd., Wuhan, China), and BHB (Cat No. JYM0071Bo, Wuhan jiyinmei Biotechnology Co., Ltd., Wuhan, China) were measured using commercial kits. Indices of cytokines were also measured, including adiponectin (Cat No. KTE10126, Abbkine Scientific Co., Ltd., Wuhan, China), leptin (Cat No. KTE10340, Abbkine Scientific Co., Ltd., Wuhan, China), TNF-α (Cat No. MBS2609886, MyBioSource, San Diego, CA, USA), and IL-6 (Cat No. MBS165083, MyBioSource, San Diego, CA, USA), using the commercial assay kits according to the manufacturer’s instructions. The intra- and inter-assay coefficients of variation for NEFA, BHB, adiponectin, leptin, TNF-α, and IL-6 were 9% and 15%, 9% and 15%, 9% and 11%, 9% and 11%, 8% and 12%, and 8% and 10%, respectively.

### 2.4. Data Processing and Analyses

The raw metabolomics data were imported into the processing software Progenesis QI (Waters Corporation, Milford, CT, USA), and then baseline filtering, peak identification and integration, retention time correction, and peak alignment were performed. Finally, a data matrix with retention time, *m/z*, and peak intensity were obtained. The positive and negative data matrix were imported into SIMCA-P + 14.1 software package (Umetri AB, Umeå, Sweden) to perform principle component analysis (PCA) and (orthogonal) partial least squares discriminant analysis (OPLS-DA). Mean centering and unit variance scaling were used before data matrix analysis. The value of variable importance in projection (VIP) score, which was greater than 1 was the typical rule for selecting differential variables in OPLS-DA analysis. To prevent overfitting, the default 7-round cross-validation was used, and the mathematical model of each round excluded one-seventh of the samples. To screen out the different metabolites between two groups, the method of *t*-test combined with multivariate analysis of OPLS-DA were used, and the difference was significant when VIP > 1.0 and *p* value < 0.05. The identification of metabolites was obtained by matching the MS and MS/MS information with the metabolic database. The identification of metabolites was obtained by matching MS and MS/MS information with publicly available databases consisting of the human metabolome database (HMDB; http://www.hmdb.ca), as well as the metabolite and tandem MS database (METLIN; http://metlin.scripps.edu). In the HMDB library and METLIN database, metabolite identification was performed with a maximum tolerance of 10 ppm mass error for fragments in the MS/MS spectra. Metabolites pathway analysis was conducted on the KEGG database (http://www.genome.jp/kegg/). The online platform of the Majorbio I-Sanger Cloud Platform (http://www.majorbio.com) was used as the data analysis tool. By default, Benjamini and Hochberg methods were used to verify the *p* value to control the false positive of the enrichment results. The corrected *p* value less than 0.05 was regarded as statistically significant. Correlations between classic metabolic parameters and newly identified metabolites were assessed by Spearman’s correlation analysis using the PROC CORR procedure of SAS 9.4.

## 3. Results

### 3.1. Plasma Metabolomics Profiling

A Total ion chromatogram of seven QC samples displayed good peak shapes and relatively uniform peak distribution in the positive and negative acquisition mode, indicating that the detection process was stable (Figure S1). In addition, the QC samples were well clustered in PCA score plots, indicating the stability of the instrument and reliability of the results (Figure S2).

**Figure 1:**
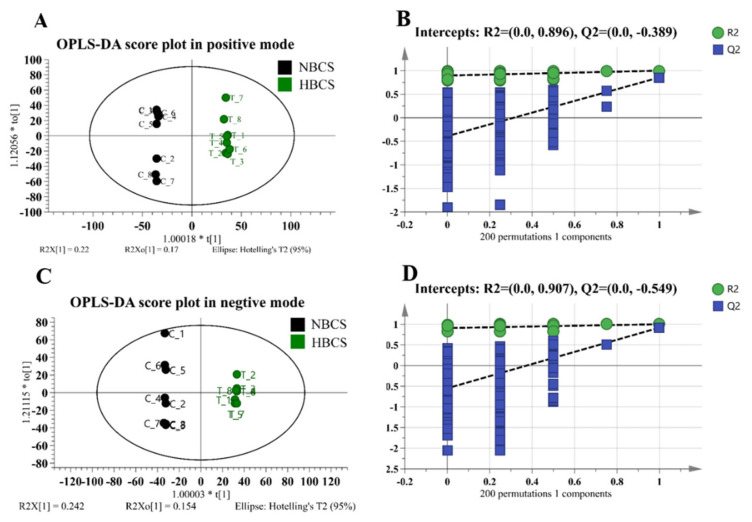
(**A**,**C**) Orthogonal partial least squares discriminant analysis (OPLS-DA) plot of plasma of cows with different calving body condition score in the positive and negative ion mode, respectively. (**B**,**D**) permutation test plots of plasma of cows with different calving body condition score in the positive and negative ion mode, respectively.

The OPLS-DA model was performed to distinguish the overall differences in metabolic profiles and to find differential metabolites between the two groups. Samples of NBCS and HBCS groups could be separated clearly without any overlap in OPLS-DA score plots under the positive and negative mode condition (Figure 1A,C). In positive iron mode, the OPLS-DA model produced one predictive component and two orthogonal components. In addition, the cumulative explained variation of R^2^X (cum) and R^2^Y (cum), and estimate of model predictive ability (Q^2^ (cum)) were 0.486, 0.999, and 0.855, respectively. Moreover, the OPLS-DA model for two groups resulted in one predictive component and three orthogonal components with R^2^X (cum) = 0.611, R^2^Y (cum) = 0.999, and Q^2^ (cum) = 0.917 in negative iron mode. The intercepts of Q^2^ in two iron modes were −0.389 and −0.549, and both were less than 0.05, indicating an absence of overfitting (Figure 1B,D).

**Figure 2:**
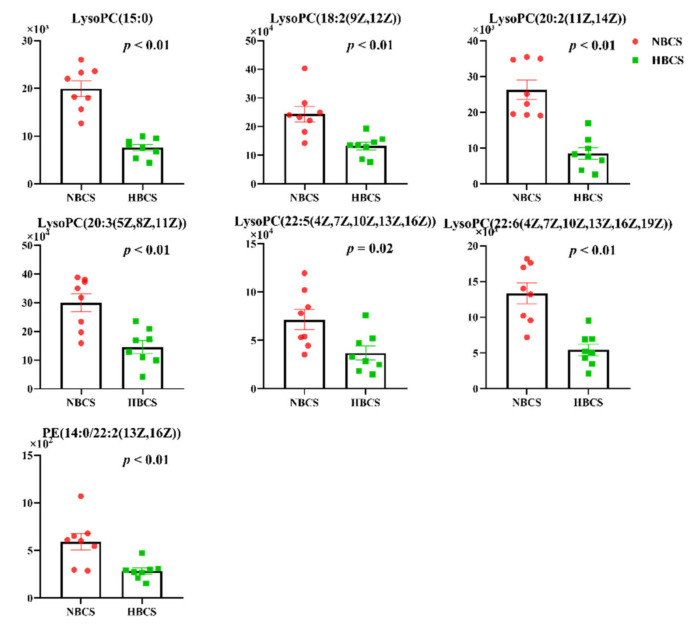
The plasma abundance of seven significantly differentiated metabolites identified by untargeted LC-MS metabolomics in cows with different calving body condition score. NBCS, normal body condition score; HBCS, high body condition score.

Based on the results of LC-MS, a total of 23 differential metabolites for distinguishing NBCS from those of HBCS group with VIP >1.0 and *p* < 0.05 are presented in Table S1. Among these metabolites, seven important metabolites identified included six distinct lysophosphatidylcholines [LysoPC(15:0), LysoPC(18:2(9Z,12Z))), LysoPC(20:2(11Z,14Z)), LysoPC(20:3(5Z,8Z,11Z)), LysoPC(22:5(4Z,7Z,10Z,13Z,16Z)) and LysoPC(22:6(4Z,7Z,10Z,13Z,16Z,19Z))], as well as a phosphatidylethanolamine (PE(14:0/22:2(13Z,16Z))). Furthermore, the concentrations of the seven metabolites were decreased in the HBCS group compared with NBCS group (Figure 2).

**Figure 3:**
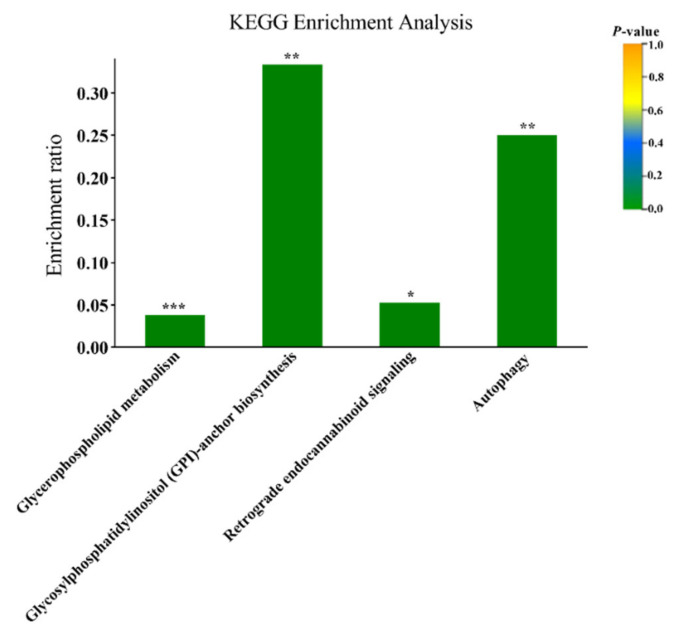
Metabolic pathway enrichment analysis of plasma of cows with different calving body condition score. The *X*-axis indicates the name and classification of the pathway. *Y*-axis represents the enrichment ratio (Enrichment ratio = sample number/background number). “***”, “**”, and “*” in the figure means the value of *p* < 0.001, < 0.01 and < 0.05, respectively. KEGG, Kyoto Encyclopedia of Genes and Genomes.

Based on the KEGG pathway analysis, they were significantly enriched in four pathways in accordance with a corrected *p* value by the Benjamini and Hochberg method (*p* < 0.05): glycerophospholipid metabolism, retrograde endocannabinoid signaling, autophagy, and glycosylphosphatidylinositol (GPI)-anchor biosynthesis (Figure 3).

### 3.2. Classical Metabolic Parameters

The classical metabolic values are shown in Table 1. The concentrations of leptin (*p* = 0.06) and TNF-α (*p* = 0.08) tended to be greater, while adiponectin (*p* = 0.09) tended to be lower in HBCS cows compared to the NBCS cows (Table 1). In addition, no difference could be found in the concentrations of NEFA, BHB, or IL-6 between the NBCS and HBCS groups expect that the numerical value of these three indexes were slightly higher in the HBCS group.

### 3.3. Correlations Analysis

**Figure 4:**
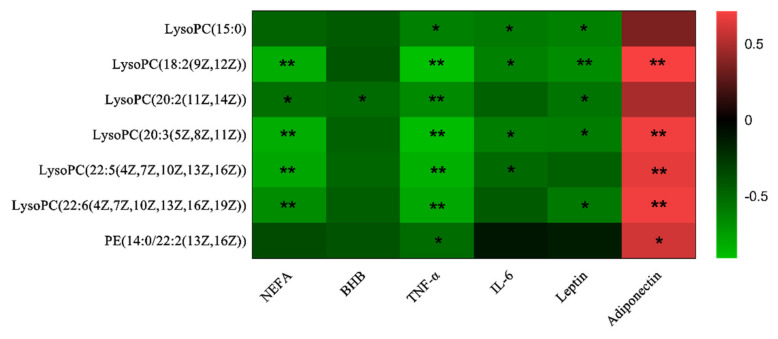
Correlations between plasma classic metabolic parameters and differential metabolites in cows with different calving body condition score. Red represents a positive correlation, while green represents a negative correlation. “**” and “*” in the figure means that the value of *p* < 0.01 and < 0.05, respectively.

According to the Spearman’s correlation analysis, the seven important metabolites seemed to be significantly correlated with plasma classic metabolic parameters (Figure 4). All these seven metabolites were negatively correlated with indices of energy metabolism [NEFA and BHB], and proinflammatory cytokines [TNF-α and IL-6]. Moreover, they were negatively correlated with leptin concentration, but positively correlated with adiponectin concentration.

## 4. Discussion

As metabolomics can provide useful information at a global system level, in the present study, the differences in the plasma metabolite profiles of dairy cows with two levels of CBCS were identified by LC-MS. In fact, seven important metabolites were identified and involved in four KEGG pathways. For our interest, the concentrations of classic metabolic indexes were also measured. A link between BCS and metabolic indicators has been discussed in obvious studies [15,16,19]. It is well known that leptin, the intake satiety signal, regulates the whole-body metabolism by stimulating energy expenditure and inhibiting food intake [3]. Adiponectin helps to enhance fatty acid oxidation and energy expenditure and inhibit proinflammatory cytokines production [20,21]. In our study, circulating leptin levels were up-regulated, and adiponectin levels were down-regulated in the HBCS group, which is similar to the results in humans [22] and rodents [23]. In addition, the concentration of TNF-α tended to be greater in HBCS cows, indicating higher calving BCS was associated with a proinflammatory response [17]. Pires et al. [16] reported that cows calving with high BCS presented elevated plasma NEFA and BHB concentrations during the early lactation (up to 7 weeks postpartum), as explained by metabolically, due to the intense mobilization of body fat. Surprisingly, the concentrations of NEFA and BHB were not affected by CBCS in this study. This may be because during the early lactation, BCS loss associated mobilization of body fat is more important in concentration of metabolic indicators rather than BCS per se [24]. It may also be due to the fact that the plasma samples were collected at the special occasion of calving, and the hormone stimulation may affect the lipid metabolism. However, the Spearman’s correlation analysis demonstrates a significant correlation between the traditional indicators and the seven metabolites, indicating these differential metabolites may be important molecules related to metabolic balance in over-condition cows.

In plasma, significant amounts of LysoPCs are formed by lecithin:cholesterol acyltransferase that catalyzes the transfer of the fatty acids of position sn-2 of PC to the free cholesterol [25,26]. LysoPCs have various species because they have different combinations of fatty acids with varying lengths and saturation. In this study, all species of LysoPCs in plasma concentrations decreased (saturated and unsaturated) in the HBCS group, which is in line with the results in humans [13], mice [12], and dogs [9]. Barber et al. [12] discovered that a high-fat diet caused a reduction in plasma LysoPC levels in mice. In addition, obese humans showed a reduction in plasma LysoPCs, indicating that adiposity plays an important role in altering the plasma LysoPC profile [12]. Moreover, the decrease of LysoPC in obesity was negatively correlated with body mass index [7]. In this study, the abundance of LysoPCs were negatively correlated with elevated energy metabolism values; therefore, the reduction of plasma LysoPCs in this study might be partly due to the slight accumulation of NEFA that could induce metabolic stress in the HBCS cows [13]. However, inconsistent with the results of our study, Pietiläinen et al. [22] reported that concentrations of LysoPCs were higher in obese co-twins compared to non-obese co-twins. To sum up, body condition changes LysoPCs metabolism profile, which is an important indicator.

As important signaling molecules, LysoPCs have diverse biological functions on cellular signaling processes, apoptosis induction, and anti-infective effects, especially on the inflammation process [27,28]. At present, the evidence is emerging to indicate that LysoPCs serve as important mediators in inflammatory diseases [28]. In this study, there was a negative correlation between LysoPCs and proinflammatory cytokines (TNF-α and IL-6), similar to the results of Heimerl et al. [7], who reported that LysoPCs correlated significantly with a proinflammatory indicator (e.g., C-reactive protein), and the decreased concentration of LysoPC was associated with its protective and anti-inflammatory effects. Thus, given fat cows being in serious proinflammatory conditions, it is reasonable to speculate that alterations of LysoPCs profile may be related to the role of LysoPCs in neutralizing the inflammation. Additionally, it was reported that LysoPCs may regulate inflammation through their fatty acyl chain [29]. For example, the unsaturated LysoPCs (20:4 and 22:6) could counteract proinflammatory effects [13,30]. Whereas saturated LysoPC (16:0), acting as an inflammatory inducer, could promote proinflammatory cytokine activity, cause migration of immune cells, and cause plasma leakage [30]. However, the reason for the decrease in saturated LysoPC(15:0) in HBCS requires further study.

In mammalian membranes, phosphatidylethanolamine (PE) is the second most abundant phospholipid, accounting for about 15–25% of the total lipids of mammalian cells [31]. In this study, PE(14:0/22:2(13Z,16Z)) differs significantly between the NBCS and HBCS groups, which not only participates in autophagy, but also in retrograde endocannabinoid signaling, glycerophospholipid metabolism, and GPI-anchor biosynthesis. Among them, autophagy, and retrograde endocannabinoid signaling pathways are closely related to obesity-associated metabolic disorders [32,33,34].

As an important component for autophagy, PE is covalently attached to the microtubule-associated protein light chain 3 to trigger autophagosome formation in mammalian cells [35]. Autophagy has been shown to regulate cellular lipid storage and lipid droplets biogenesis/breakdown [33,36]. Decreased autophagy contributes to hepatic lipid accumulation and then further inhibits autophagic function, thereby increasing lipid retention [36,37]. In addition, excessive lipid infiltration in the liver of cows impairs autophagic activity [34]. Since PE(14:0/22:2(13Z,16Z)) was negatively correlated with NEFA and BHB, the decrease of PE(14:0/22:2(13Z,16Z)) concentration in the HBCS group in this study may contribute to the suppression of autophagy and then promote excessive lipids accumulation. In support of this, Ghaffari et al. [38] have shown that autophagy in HBCS cows may be suppressed, because they found that the mRNA abundance of mammalian target of rapamycin (mTOR) in the skeletal muscle was greater in high BCS cows than in normal BCS cows on d + 21 after calving, and mTOR was considered to be a potent suppressor of autophagy [39,40].

PE participates in the synthesis of anandamide, which is an endogenous ligands of cannabinoid receptors in the retrograde endocannabinoid signaling pathway [41]. It has been reported that the endocannabinoid system is involved in regulating energy balance, such as feed intake, energy expenditure, and lipid metabolism [42]. In addition, the formation of anandamide is accompanied by the production of ethanolamides of other fatty acids, which are collectively referred to as N-acylethanolamines (NAEs) [43]. However, major NAEs do not show ligand activity for cannabinoid receptors, but they participate in a variety of biological activities. In particular, oleoyl-ethanolamide (OEA, a non-endocannabinoid NAE) mediates the satiety and the bodyweight through activation of the peroxisome proliferator-activated receptor-α [44], a nuclear receptor that promotes fatty acid oxidation and reduces triglyceride level. The dysfunction of OEA signaling could contribute to overweight and obesity [45]. In this study, PE(14:0/22:2(13Z,16Z)) was involved in the retrograde endocannabinoid signaling pathway, but its exact role in this pathway remains to be further clarified. In the present study, the number of cows used was limited. Thus, studies with more individuals should be conducted to better understand the plasma metabolic changes in cows with different body conditions.

## 5. Conclusions

Combined with the measurement of classic metabolic indexes, this study provides new insight into the depth of metabolic changes that occur in the plasma of over-conditioned fresh cows. Seven important differential metabolites were identified, including six distinct LysoPCs and a PE. Moreover, these seven metabolites are closely related to lipid metabolism, inflammation, and over-conditioning in cows, and may provide a metabolic basis for the diseases associated with over-conditioned dry cows.

## Figures and Tables

**Table 1:** Classical metabolic parameters of plasma of cows with different calving body condition score.

Items	Normal Body Condition Score (*n* = 8)	High Body Condition Score (*n* = 8)	SEM	*p*-Value
Nonesterified fatty acid, nmol/mL	162.6	171.0	12.77	0.52
β-hydroxybutyrate, µg/mL	9.97	11.56	0.916	0.10
Leptin, pg/mL	1840.4	2078.4	116.5	0.06
Adiponectin, ng/mL	10.02	8.67	0.757	0.09
Tumor necrosis factor –α, pg/mL	85.2	99.4	7.58	0.08
Interleukin 6, pg/mL	94.6	104.0	7.12	0.21

## Supplementary Materials

The following are available online at https://www.mdpi.com/2076-2615/10/9/1709/s1, Figure S1: UPLC-Triple TOF total ion chromatograms of the plasma QC samples, Figure S2: The PCA of LC-MS of the plasma QC samples corresponding to different calving body condition score, Table S1: Identification of significantly different metabolites in plasma in cows with different calving body condition score.
