# Supplementary material for: Metabolic Profiling of Plasma in Different Calving Body Condition Score Cows Using an Untargeted Liquid Chromatography-Mass Spectrometry Metabolomics Approach
Source: Animals (Basel). 2020 Sep 21;10(9):1709. doi: 10.3390/ani10091709 (PMC7552654; doi:10.3390/ani10091709)
Supplement: Supplementary file 1 [file animals-10-01709-s001.pdf]

animals

Supplementary Materials: Metabolic Profiling of Plasma in Different Calving Body Condition Score Cows Using an Untargeted Liquid Chromatography-Mass Spectrometry Metabolomics Approach

Jian Wang, Chuang Zhang, Qingyao Zhao, Congcong Li, Shuang Jin and Xianhong Gu *

State Key Laboratory of Animal Nutrition, Institute of Animal Sciences, Chinese Academy of Agricultural Sciences, Beijing 100193, China; wangjian_1884@163.com (J.W.); 18241653656@163.com (C.Z.); zhaoqingyao63@163.com (Q.Z.); congcongli1988@sina.com (C.L.); shuangjinjs@163.com (S.J.)

* Correspondence: guxianhong@caas.cn

Received: 04 August 2020; Accepted: 15 September 2020; Published: date

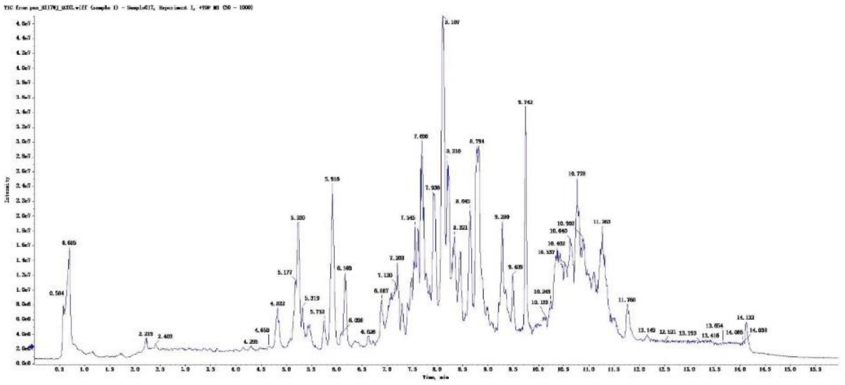

(a)

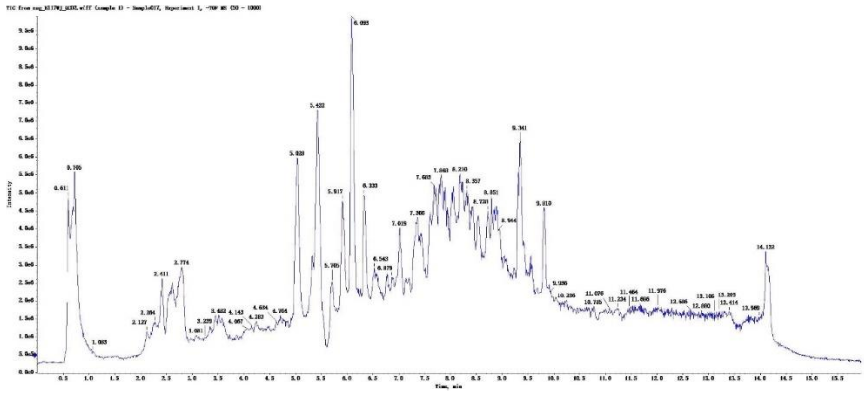

(b)

Figure S1. UPLC-Triple TOF total ion chromatograms of the plasma QC samples. (**a**) Total ion chromatograms of QC samples in the positive ion mode. (**b**) Total ion chromatograms of QC samples in the negative ion mode.

UPLC-Triple TOF, ultra-high pressure liquid chromatography coupled with a Triple quadrupole time-of-flight; QC, quality control.

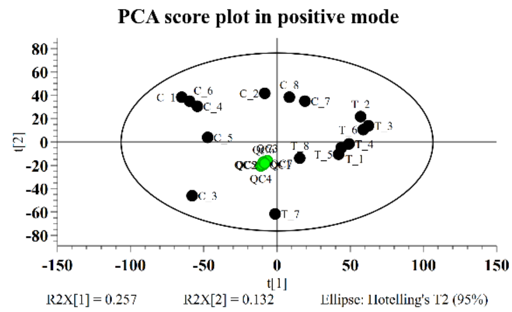

(a)

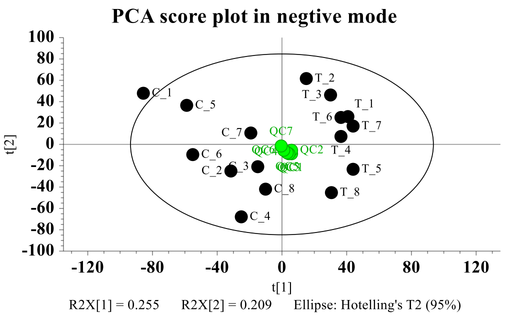

(b)

Figure S2. The PCA of LC-MS of the plasma QC samples corresponding to different calving body condition score. (**a**) PCA of QC samples in the positive ion mode. (**b**) PCA of QC samples in the negative ion mode. The black circle represents a plasma sample, while green circle represents a QC sample.

PCA, principal component analysis; QC, quality control.

Table S1. Identification of significantly different metabolites in plasma in cows with different calving body condition score.

Metabolite name	Mass Error (ppm)	m/z	RT (min)	Ion mode	VIP	Fold change (NBCS/HBCS)	*P*- value
LysoPC(15:0)	1.75	482.3	7.57	pos	1.89	2.63	< 0.01
LysoPC(18:2(9Z,12Z))	1.74	520.3	7.55	pos	1.52	1.85	0.0024
LysoPC(20:2(11Z,14Z))	1.87	548.4	8.33	pos	1.79	3.08	< 0.01
LysoPC(20:3(5Z,8Z,11Z))	1.57	546.4	7.92	pos	1.56	2.06	0.0014
LysoPC(22:5(4Z,7Z,10Z,13Z,16Z))	1.36	570.4	7.74	pos	1.26	1.93	0.0169
LysoPC(22:6(4Z,7Z,10Z,13Z,16Z,19Z))	-1.60	568.3	7.51	pos	1.68	2.44	0.0003
PE(14:0/22:2(13Z,16Z))	-5.19	788.5	10.62	neg	1.35	2.08	0.0051
1-heptadecanoyl-sn-glycero-3-phosphocholine	2.72	510.4	8.45	pos	1.50	1.78	0.0027
1-Linoleoylglycerophosphocholine	1.23	520.3	7.68	pos	1.49	1.41	0.0031
3'-N'-Acetylfusarochromanone	4.50	299.1	3.74	pos	1.25	0.26	0.0174
Asperagenin	2.33	449.3	6.90	pos	1.36	0.54	0.009
LysoPC(0:0/18:0)	2.39	524.4	8.65	pos	1.18	1.48	0.03
LysoPC(20:0)	1.86	552.4	8.87	pos	1.79	2.37	< 0.01
PC(20:5(5Z,8Z,11Z,14Z,17Z)/0:0)	0.41	542.3	7.16	pos	1.58	2.39	0.0011
PC(7:0/O-8:0)	2.46	482.3	7.73	pos	1.68	1.89	0.0003
(-)-3-Cyanomethyl-3-hydroxy-1H-indol-2(3H)-one	-9.67	375.1	3.69	neg	1.71	0.35	< 0.01
4'-O-methyl-(-)-epicatechin-5-O-beta-glucuronide	-4.55	459.1	4.45	neg	1.57	0.26	0.0004
5-Hydroxyflavone	-9.99	445.1	3.63	neg	1.54	0.23	0.0005
Amphibine H	3.31	586.3	7.29	neg	1.34	2.11	0.0052
Dolicholide	-6.89	513.3	8.41	neg	1.43	1.83	0.0023
PtdIns-(3)-P1 (1,2-dioctanoyl) (sodium salt)	5.15	665.2	4.23	neg	1.58	0.23	0.0004
R-95913	-4.24	376.1	4.63	neg	1.46	0.49	0.0019
Ustiloxin A	-9.40	694.2	5.50	neg	1.60	0.28	0.0003

RT, retention time; VIP, variable importance in the projection; Fold change, ratio of mean peak area of the normal body condition score group to the mean peak area of the high body condition score group. Mass Error in ppm, the difference between a theoretical *m*/*z* and an experimentally observed *m*/*z*.
